# Taurine effects on Bisphenol A-induced oxidative stress in the mouse testicular mitochondria and sperm motility

**DOI:** 10.5935/1518-0557.20200017

**Published:** 2020

**Authors:** Fatemeh Rezaee-Tazangi, Leila Zeidooni, Zeinab Rafiee, Fereshtesadat Fakhredini, Heybatollah Kalantari, Hadis Alidadi, Layasadat Khorsandi

**Affiliations:** 1 Student Research committee, Ahvaz Jundishapur University of Medical Sciences, Ahvaz, Iran; 2 Department of Anatomical Sciences, Ahvaz Jundishapur University of Medical Sciences, Ahvaz, Iran; 3 Toxicology Research Center, Ahvaz Jundishapur University of Medical Sciences, Ahvaz, Iran; 4 Cellular and Molecular Research Center, Ahvaz Jundishapur University of Medical Sciences, Ahvaz, Iran

**Keywords:** mitochondria, sperm motility, taurine, oxidative stress, bisphenol A

## Abstract

**Objectives:**

This study was performed to investigate the protective effects of taurine (2-aminoethanesulfonic acid, TAU) on oxidative stress in the isolated mouse testicular mitochondria, mitochondrial membrane potential (MMP), viability and motility of the exposed sperms to the BPA.

**Methods:**

We treated epididymal spermatozoa obtained from mice and isolated mouse testicular mitochondria with BPA (0.8 mmol/mL) and various doses of TAU (5, 10, 30 and 50 µmol/L). We used the MTT assay and Rhodamine 123 uptake to assess sperm viability and MMP. We assessed the oxidative stress through measuring ROS (reactive oxygen species), MDA (malondialdehyde), GSH (glutathione), and SOD (super-oxide dismutase) levels in the testicular mitochondrial tissue.

**Results:**

BPA significantly elevated ROS, MDA and MMP levels, and markedly reduced SOD and GSH levels in the isolated mitochondria. BPA also considerably impaired spermatozoa viability and motility. Pretreatment with 30 and 50 µmol/L of TAU could considerably suppressed mitochondrial oxidative stress, enhanced MMP, and improved sperm motility and viability.

**Conclusion:**

TAU may attenuate the BPA-induced mitochondrial toxicity and impaired sperm motility via decreasing oxidative stress.

## INTRODUCTION

Bisphenol A (BPA), a polycarbonate plastic and a constituent of epoxy and polystyrene resins, is used in coatings of beverages, food cans, and baby bottles, and it is used in thermal containers, dental sealants, and medical devices (Vandenberg *et al*., 2007; Mikołajewska *et al*., 2015; Anjum *et al*., 2011). The migration of BPA into the environment depends on pH and temperature (Scippo, 2011; Braun *et al*., 2011). BPA enters the body via dermal contact, inhalation and ingestion (Siracusa *et al*., 2018). The human exposure to BPA depends on the BPA levels in the environment, biological systems, and food intake. There can be BPA in semen, urine, plasma, breast milk and amniotic fluid (Engel *et al*., 2014; Ye *et al*., 2006; Ikezuki *et al*., 2002).

BPA has toxic impacts on various tissues, including the male reproductive system (Ullah *et al*., 2018; Anjum *et al*., 2011). BPA can reduce testicular and epididymal weights in rodents (Chitra *et al*., 2003) and impair sperm quality (Li *et al*., 2016). In addition, BPA induces mitochondrial dysfunction by reducing ATP, diminishing the mass of mitochondria, and disrupting membrane potential (Kaur *et al*., 2014; Lin *et al*., 2013). Mitochondrial dysfunction can affect sperm motility and sperm production (Chattopadhyay *et al*., 2010). Moreover, BPA suppresses antioxidant activity and enhances ROS production in rat testicles (Chitra *et al*., 2003).

Taurine (TAU), is a free amino acid, present in several mammalian tissues such as the reproductive system (De Luca *et al*., 2015; Park *et al*., 2002). It has several physiological functions, including energy storage, membrane stabilization, xenobiotic conjugation, and antioxidation (Huxtable, 1992). There is TAU in seminal fluid, vascular endothelial cells, germinal cells, Leydig cells and in the covering epithelium of efferent ducts (Holmes *et al*., 1992; Hinton, 1990). TAU may act as an antioxidant, membrane-stabilizing and motility factors of the sperm (Yang *et al*., 2015). The current research investigated TAU impacts on BPA-induced mitochondrial oxidative stress and impaired sperm motility in mice.

## MATERIALS AND METHODS

### Experimental design

We collected sperm samples and isolated testicular mitochondria from forty-two adult NMRI mice (8-10 weeks). The Ethics Committee on Animal Research confirmed this study (No: ABHC.REC.1397.079).

**Figure 1:**
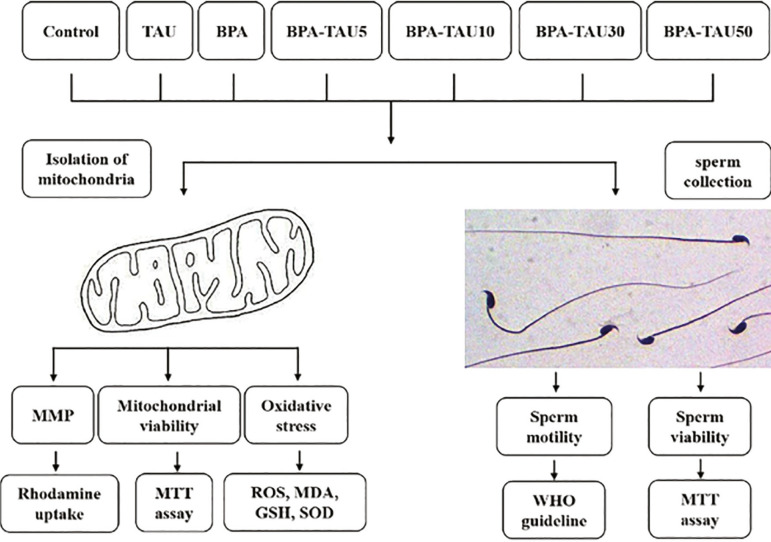
Schematic illustration of experimental design

We obtained the spermatozoa from the epididymis, as per previously described (Su *et al.,* 2019), and categorized into the following groups (Figure 1). In each group, we used 5× 10^6^sperm/ml (Harris *et al*., 2007).

1. Control: received only media
2. BPA: exposed to 0.8 mmol/ L BPA for 2 hours
3. TAU: exposed to 50 µmol/ L TAU for 4 hours
4. BPA+TAU5: pretreated with 5 µmol/ L of TAU for 2 hours before BPA treatment (2 hours).
5. BPA+TAU10: pretreated with 10 µmol/ L of TAU for 2 hours before BPA treatment (2 hours).
6. BPA+TAU30: pretreated with 30 µmol/ L of TAU for 2 hours before BPA treatment (2 hours).
7. BPA+TAU50: pretreated with 50 µmol/ L of TAU for 2 hours before BPA treatment (2 hours).

We kept all samples at 37ºC in an incubator during the experiment. The untreated sperms (control groups) began to die after 4 hours. Hence, 4 hours were used to treat the sperms with TAU and BPA. BPA (Sigma) was dissolved in 0.1% dimethyl sulfoxide (DMSO, Sigma) and then diluted in media (ham's F10, Invitrogen). The BPA dose was chosen according to the IC_50_ value (Table 1). To determine the IC_50_ of BPA, the sperm viability was determined using an MTT assay. We dissolved the TAU in distilled water and stored at 4ºC until use.

**Table 1:** The IC_50_ (µM) of BPA on the spermatozoa

Concentrations	1 hour	2 hours
100 (µmol L^-1^)	98.7±4.35	95.4±6.23
200 (µmol L^-1^)	91.9±5.11	85.6±4.31
400 (µmol L^-1^)	76.2±5.65	66.1±4.37
800 (µmol L^-1^)	64.1±4.75	49.5±3.98
1000 (µmol L^-1^)	55.2±3.63	38.3±3.55

Values are expressed as mean ± SD (n=6).

### Mitochondria isolation

The mice testicles were removed under deep anesthesia and minced in a cold isolating medium which contained EDTA (0.1 mmol, Sigma), EGTA (0.2 mmol, Sigma), sucrose (250 mmol, Sigma), HEPES-KOH (5 mmol, Sigma) and 0.1% fat free BSA (bovine serum albumin, Invitrogen). The minced blood-free testicles were homogenized and centrifuged at 3000·g for 7 minutes (at 4ºC). The supernatant was centrifuged at 10,000·g for 7 minutes. The obtained pellet (mitochondrial fraction) was suspended and pelleted twice at 10,000·g for 10 minutes. After washing, the protein content was determined using the Bradford assay reagent (Bio-RAD). We divided the isolated mitochondria into 7 groups, similar to the sperm groups, and the mitochondrial fractions (0.5 mg protein/mL) were exposed to the similar concentration and duration time of BPA and TAU.

### MTT assay

The isolated mitochondria or sperms were placed in a 96 well plate and treated with BPA or TAU. Ten µL of MTT (Sigma, USA) at concentration of 5 mg/mL media was poured into each well and incubated at 37ºC for one hour. When the media was removed, 100 µL of DMSO was poured into the wells. Finally, the absorbance at 570 nm was determined using a micro-plate reader.

### Determining MDA content, ROS level and anti-oxidant enzyme activity

After treatment, we poured the isolated mitochondria samples (1 mL) into the micro-tubes. We removed the media and added 10 µmol of DCFH-DA (Sigma) and 100 µl of Hank's buffered salt solution (Invitrogen) at 37ºC for 30 minutes. We measured ROS levels using a spectro-fluorometer (LS50B, USA, Ex: 490 nm, Em: 570 nm). After treatment, we identified the protein contents of the isolated mitochondria using a BCA protein assay kit (Pierce Biotechnology Inc. IL). After centrifuging, we evaluated the malondialdehyde (MDA) content, and the level of GSH (glutathione) and superoxide dismutase (SOD) according to the kit's instruction (ZellBio Company).

### Mitochondrial membrane potential (MMP) evaluation

After treatment, we exposed the fractions of mitochondria (0.5 mg protein/mL) to ten µmol of Rhodamine 123 for 15 minutes. We measured the fluorescence using a spectrophotometer (LS50B, USA; excitation: 490 nm; emission: 535 nm).

### Sperm motility

We assessed sperm motility according to the WHO guidelines (Su *et al*., 2019), using ten µL of sperm suspension poured into a semen analysis chamber. We evaluated five microscopic fields to estimate sperm motility on at least 200 spermatozoa for each sample, assessing the percentage of sperm motility using the following motion patterns: fast progressive (A), slow progressive (B) no progressive C) and immotile sperms (D).

### Statistical Analysis

We analyzed the data using the SPSS (version 21.0, employing one-way analysis of variance, post-hoc test, and Bonferroni correction. In addition, the *p*-value <0.05 was considered significant.

## RESULTS

### Viability

**Figure 2:**
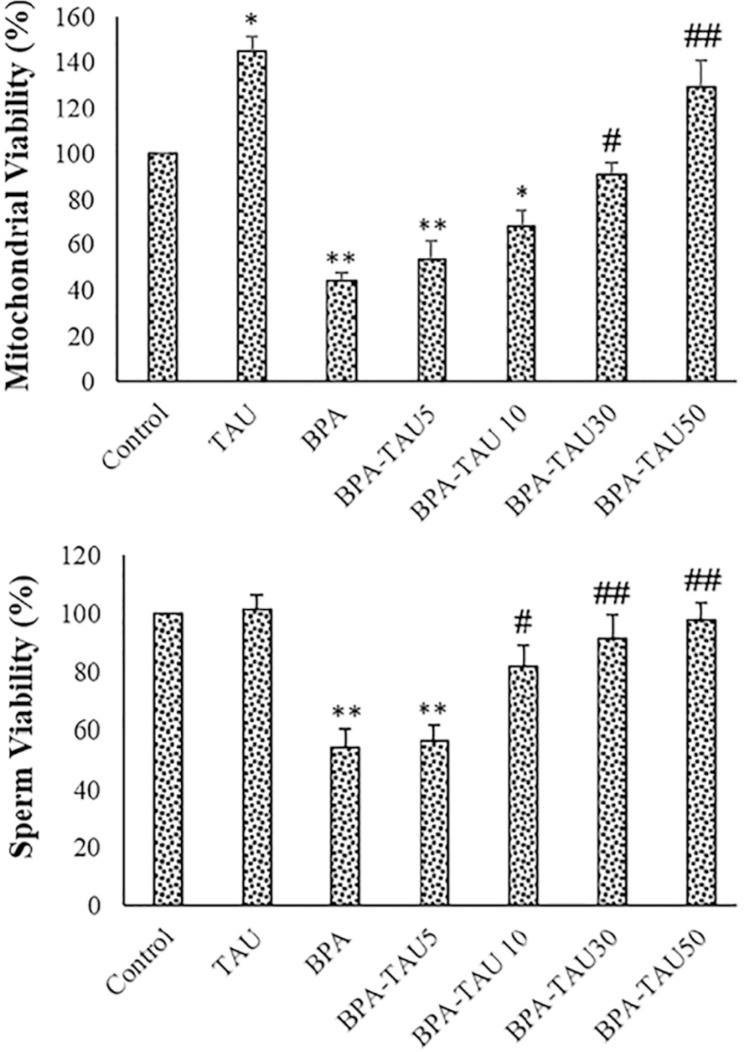
Viability percentage of the isolated mitochondria and sperms. The mean ± standard deviations are shown (n=6). * *p*<0.05, ** *p*<0.01, # *p*<0.05, ## *p*<0.01; * and # symbols show comparison to the control and BPA groups, respectively.

As reported in Figure 2, following BPA exposure, viability percentage significantly reduced in the isolated testicular mitochondria and spermatozoa (*p*<0.01). The viability percentage significantly increased in the TAU-exposed mitochondria (*p*<0.05). TAU at the doses of 30 and 50 µmol/L reversed the viability of the BPA-exposed sperms and the testicular mitochondria. DMSO did not significantly affect sperm viability and motility (Table 2).

**Table 2:** DMSO effects on isolated mitochondria and sperms

Parameters	Control	DMSO
Sperm viability of (%)	100±0.00	100.2±1.23
Mitochondria viability (%)	100±0.00	99.6±1.16
MMP (% of control)	100±0.00	100.05±0.94
ROS formation (% of control)	100±0.00	98.7±2.35
Mitochondria MDA (nmol/ mg protein)	18.2±5.65	17.9±3.36
Mitochondria GSH (pmol/ mg protein)	11.51±2.75	49.5±3.98
Mitochondria SOD (U/ mg protein)	10.28±2.65	9.92±2.16
Total sperm motility (%)	69.85±5.78	71.32±6.45

Values are expressed as mean ± SD (n=6).

### ROS measurement

**Figure 3:**
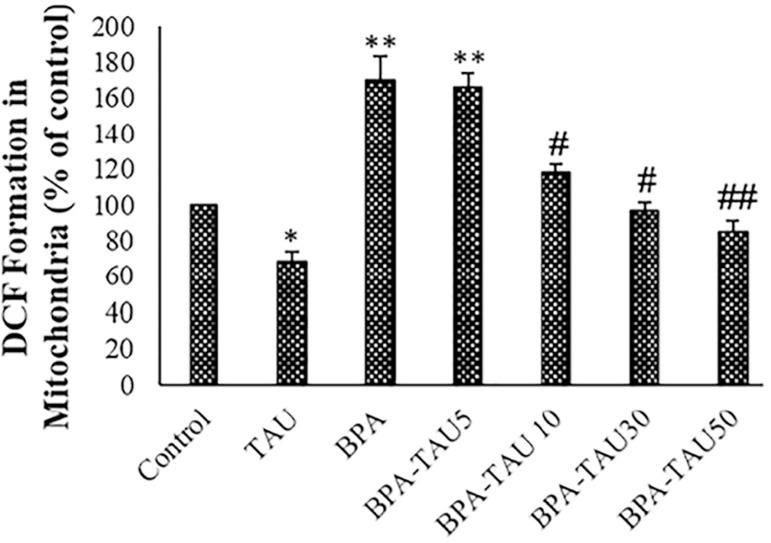
DCF formation (ROS levels) in the spermatozoa and isolated mitochondria. The mean ± standard deviations are shown (n=6). * and # symbols show a comparison of the control and BPA groups, respectively.

In the BPA group, the ROS generation was considerably elevated in the testicular mitochondria (*p*<0.01). ROS generation was significantly reduced in the TAU treated samples in comparison with the control. TAU dose-dependently attenuated ROS production by BPA in the testicular mitochondria (Figure 3). DMSO had no significant impact on ROS formation in comparison with the control (Table 2).

### MDA, SOD and GSH levels

**Figure 4:**
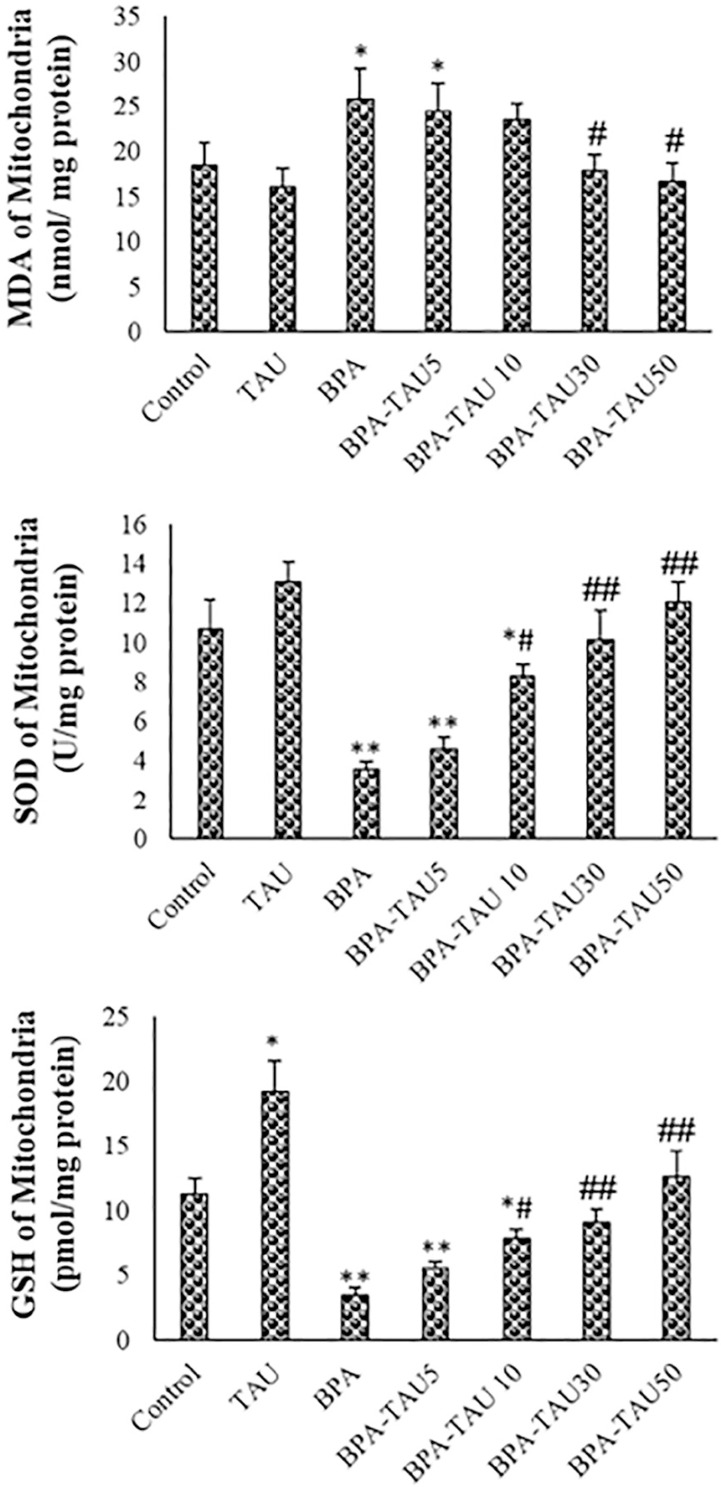
MDA, SOD and GSH levels of mice spermatozoa. The mean ± standard deviations are shown (n=6). MDA, SOD and GSH levels of the isolated mitochondria. The mean ± standard deviations are shown (n=6). * and # symbols show a comparison of the control and BPA groups, respectively.

Following BPA exposure, MDA levels were significantly increased in the isolated testicular mitochondria compared to the control (*p*<0.01). MDA levels were slightly reduced in the TAU-treated mitochondria in comparison with the control. At the doses of 10, 30 and 50 µmol/L, TAU attenuated BPA increased MDA levels in the testicular mitochondria. SOD and GSH levels were considerably elevated in the BPA-exposed mitochondria (*p*<0.01). Following TAU treatment, SOD levels were slightly increased while GSH levels were significantly elevated, compared to the control. In a dose-dependent fashion, TAU attenuated BPA- reduced antioxidant activity in the testicular mitochondria (Figure 4). DMSO had no significant impact on MDA, SOD and GSH levels in the mitochondria (Table 2).

### MMP Assay

**Figure 5:**
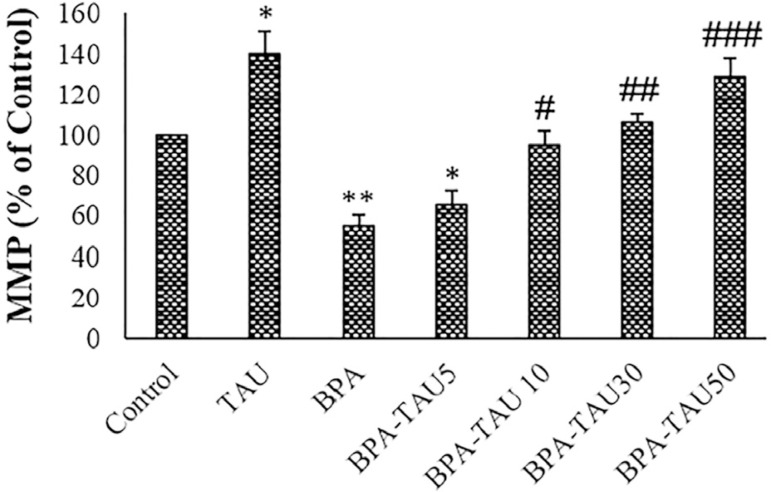
MMP measurement in the control and experimental groups. The mean ± standard deviations are shown (n=6). ### *p*<0.001; * and # symbols show a comparison of the control and BPA groups, respectively.

As reported in Figure 5, TAU significantly increased MMP in the testicular mitochondria (*p*<0.05). Following BPA exposure, MMP was significantly reduced compared to the control (*p*<0.01). TAU at the doses of 10, 30 and 50 µmol/ L effectively enhanced the MMP of the BPA-treated mitochondria. DMSO had no significant impacts on the MMP in comparison to the control (Table 2).

### Sperm motility

**Figure 6:**
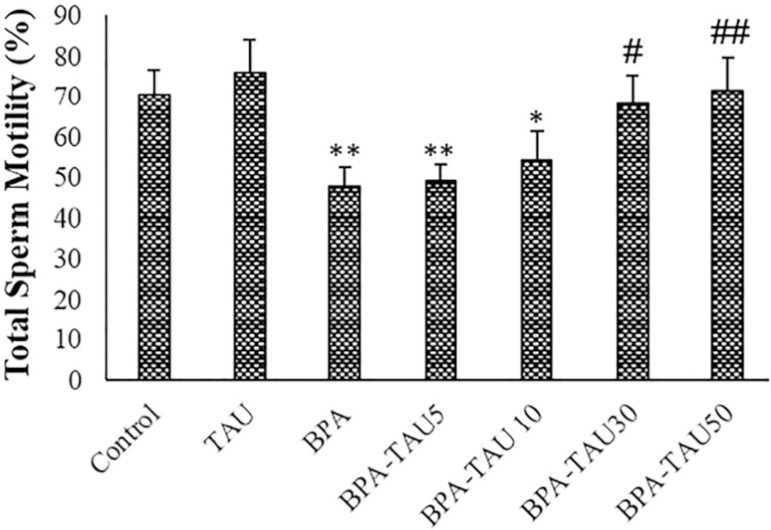
Total sperm motility in the different groups. The mean ± standard deviations are shown (n=6). * and # symbols show a comparison to the control and BPA groups, respectively.

TAU slightly increased total sperm motility in comparison to the control. Following BPA exposure, total sperm motility (*p*<0.01) and fast progressive sperm percentages (*p*<0.05) were significantly reduced, while immotile sperm percentage was markedly increased (*p*<0.01). TAU dose-dependently reversed the total sperm motility, fast progressive sperm percentages, and the percentage of immotile sperms (Table 3 and Figure 6). DMSO had no significant impacts on sperm motility when compared to the control (Table 3).

**Table 3:** Velocity distribution of spermatozoa in different groups

Groups	Fast progressive	Slow progressive	No progressive	Immotile
Control	39.77±4.11	30.27±3.83	17.11±2.46	12.85±2.86
TAU	42.54±3.36	33.62±4.18	16.17±2.53	7.67±1.12*
BPA	25.77±2.27*	21.85±2.14*	22.58±3.25	29.17±2.67*
BPA-TAU2.5	31.97±3.15	19.25±2.19	24.95±2.71	25.67±3.12*
BPA-TAU5	32.53±4.2	21.62±3.11	22.92±3.33	22.93±2.88*
BPA-TAU10	36.15±4.5#	31.87±4.21	21.67±3.52	10.31±1.89
BPA-TAU20	41.26±5.1##	30.25±3.92	20.15±2.91	8.34±1.12##

The mean ± standard deviations are shown (n=6). * *p*<0.05, # *p*<0.05, ## *p*<0.01; * and # symbols show comparison to the control and BPA groups, respectively.

## DISCUSSION

Our study showed that TAU reversed the viability and motility of the BPA-exposed sperms in a dose-dependent fashion. Previous reports showed that BPA caused a decrease in sperm quality in rodents and humans (Rahman *et al*., 2016; Wisniewski *et al*., 2015; Kotwicka *et al*., 2016). BPA impaired reproduction and sperm function in zebrafish (Chen *et al*., 2017). BPA decreased the viability of the mouse spermatocyte (Qian *et al*., 2015).

In this study, TAU dose-dependently improved viability, motility and progressive movement velocity of BPA-treated mouse sperms. In agreement with our results, Yang *et al*. (2017) reported that TAU effectively protects GC-2 (spermatocytes) cells from ionizing radiation. TAU dose-dependently enhanced sperm quality in donkeys (Bottrel *et al*., 2018). Positive effects of TAU on boar semen quality have also been reported (Li *et al*., 2016; Kutluyer *et al*., 2016). Conversely, BPA has no impact on fowl sperm motility (Barna *et al*., 1998), and it has no positive effects on the viability of short-term (4 hours) stored rabbit spermatozoa (Paál *et al*., 2017). The difference in treatment duration or species variety may represent the reasons for these contradictory results.

The mechanism of TAU action on sperm viability and motility was not elucidated in the current study. It is possible that TAU improves sperm viability via suppression of cell death signaling. Aly & Khafagy (2014) showed the anti-apoptotic effects of TAU against endosulfan in adult rat testicles. TAU inhibited apoptosis in Thiopurine-induced testicular damages in rats (Ramadan *et al*., 2018). Improved sperm motility may be due to TAU impacts in mitochondrial mass or function.

It has been reported that TAU exists in the mitochondrial matrix and membranes of various cells (Jong *et al*., 2010; Alvarez & Storey, 1995; Hansen *et al*., 2010; Shetewy *et al*., 2016). Mitochondria has a TAU transporter in its plasma membrane to uptake TAU from culture media (Suzuki *et al*., 2002). Thus, adding TAU to culture media may enhance its concentration in the mitochondria and improve mitochondrial function.

The disrupted sperm movement can also be due to high ROS levels (Barbonetti *et al*., 2016). According to our results, BPA enhanced ROS and MDA levels in the spermatozoa. In agreement with these findings, BPA enhanced ROS generation and MDA contents in the spermatozoa (Kaur *et al*., 2018; Yang *et al*., 2013; Yang *et al*., 2017; Rahman *et al*., 2019).

The present study has shown that TAU reversed ROS generation, MDA level, antioxidant factors, and MMP in the BPA-exposed mouse testicular mitochondria. Therefore, TAU may protect mitochondria by reducing oxidative stress. Consistent with our results, TAU had protective impacts on mitochondrial oxidative damage in various pathological conditions. TAU improves the function of heart mitochondria and prevents oxidative stress in diabetic rats (Gorbenko *et al*., 2016). TAU inhibits mitochondrial oxidative damage induced by Tamoxifen in the mouse liver (Parvez *et al*., 2008).

The TAU-reversed oxidative stress induced by BPA was accompanied by increasing sperm motility and viability. In the study of Minamiyama *et al*. (2010), BPA-decreased sperm motility was reversed by co-administering n-acetylcysteine. Wisniewski *et al*. (2015) demonstrated that TAU elevated anti-oxidation of the testis and enhance sperm quality.

According to our results, BPA diminished the MMP of the isolated mouse testicular mitochondria, and TAU dose-dependently reversed this event. BPA decreased the MMP and increased cell death in human spermatozoa (Barbonetti *et al*., 2016). MMP was positively correlated with total sperm number and progressive sperm motility (Zhang *et al*., 2016).

The BPA reduced MMP was accompanied by the induced mitochondrial oxidative stress and impaired sperm motility. BPA is reported to cause oxidative stress in the mitochondria obtained from testicles, leading to an elevation in lipid peroxidation (del Hoyo *et al*., 2010). Lipid peroxidation, in turn, can disrupt spermatozoa functions (Catalá, 2009).

Lipid peroxidation in mitochondria can be reversed by TAU administration (Parvez *et al*., 2008). TAU could prevent manganese-induced mitochondrial damages in isolated mice brain mitochondria (Ahmadi *et al*., 2018).

## CONCLUSIONS

In summary, TAU dose-dependently decreased mitochondrial oxidative stress and improved MMP. In addition, TAU improved the viability and motility of mice sperm. TAU can ameliorate BPA‐induced mitochondrial toxicity and impaired sperm quality by suppressing oxidative stress.
